# Imported Lassa fever: a report of 2 cases in Ghana

**DOI:** 10.1186/s12879-015-0956-2

**Published:** 2015-05-29

**Authors:** Nicholas N.A. Kyei, Mark M. Abilba, Foster K. Kwawu, Prince G. Agbenohevi, Joseph H.K. Bonney, Thomas K. Agbemaple, Shirley C. Nimo-Paintsil, William Ampofo, Sally-Ann Ohene, Edward O. Nyarko

**Affiliations:** 37 Military Hospital, Neghelli Barracks, Accra, Ghana; Noguchi Memorial Research Institute for Medical Research, University of Ghana, P.O. Box LG 581, Legon, Ghana; World Health Organization Ghana Country Office, P. O Box MB 142, Accra, Ghana

**Keywords:** Lassa fever, Viral hemorrhagic fevers, *Mastomys*, Emerging infectious disease, West Africa

## Abstract

**Background:**

Lassa fever is a potentially fatal acute viral illness caused by Lassa virus which is carried by rodents and is endemic in some West African countries. Importation of emerging infections such as Lassa fever, Ebola Virus Disease and other viral hemorrhagic fevers into non endemic regions is a growing threat particularly as international travel and commitments in resolving conflicts in endemic countries in the West Africa sub-region continue.

**Case presentation:**

We report the first two recorded imported cases of Lassa fever among Ghanaian Peace keepers in rural Liberia, who became ill while on Peace keeping mission. They were subsequently evacuated to the UN level IV hospital in Accra, where their illnesses were laboratory confirmed. One of the patients recovered with ribavirin treatment and supportive therapy. No secondary clinical cases occurred in Ghana.

**Conclusions:**

Healthcare providers at all levels of care should thus have a high index of suspicion for these infectious diseases and adopt standard infection control measures when treating patients in endemic regions or returning travelers from an endemic region with a febrile illness even of a known etiology.

## Background

Lassa fever is an old age acute viral haemorrhagic illness caused by Lassa virus, a rodent-borne arenavirus which was discovered in 1969. The single-stranded RNA virus is named after the town in Nigeria where the first cases were isolated [1, 2]. The animal reservoir and vector for Lassa fever is the “multimammate rat” (*Mastomys natalensis*) [1, 3]. It is transmitted to humans from contact with food or household items contaminated with rodent excreta. Aerosol or airborne transmission may also occur during cleaning activities, such as sweeping, when a person inhales tiny air particles contaminated with infected rodent excreta. Person-to-person infections and laboratory transmission is also possible, particularly in the absence of adequate infection control measures in the healthcare environment [1, 2]. The virus can be isolated in body fluids of infected persons including semen and secretion of the virus can continue for 30 days and beyond from infected individuals [2].

Lassa fever occurs in both sexes and all age groups. Classically signs and symptoms of the disease occur 6–21 days after a person comes into contact with the virus [1, 2]. Approximately 80 % of Lassa fever cases present with mild symptoms such as mild fever, general malaise, weakness, and headache, and are often undiagnosed. In the remaining 20 % of infected individuals however, the disease may progress to more serious symptoms including high grade fever, sore throat, mucosal bleeding (from gums, eyes, or nose, vagina as examples), respiratory distress, repeated vomiting, facial swelling, pain in the chest, back, and abdomen, and shock. Neurological problems have also been described, including hearing deficit, seizures, meningitis and encephalitis. Approximately 15–20 % of patients hospitalized for Lassa fever die from the illness. The case-fatality rate may reach 50 % in hospitalized patients during occasional Lassa fever epidemics. Generally however, only 1 % of all Lassa fever cases result in death [1, 2].

The disease is endemic in parts of West Africa particularly Sierra Leone, Liberia, Guinea and Nigeria [4]. It is estimated that between 300,000 and 500,000 Lassa fever cases and 5000 deaths occur annually in West Africa, indicating a high burden and impact on populations in these endemic regions [1, 2]. In some areas of Sierra Leone and Liberia, 10–16 % of annual hospital admissions are due to Lassa fever [1]. Neighboring countries however, remain at risk [4] as the rodent vector for Lassa virus is ubiquitous throughout the region. Sporadic Lassa fever cases have been detected in Côte d’Ivoire [5] and from travelers to Burkina Faso [6, 7]. There has been serologic evidence of Lassa virus infection in Ghana [8] as well as in Togo and Benin [9]. In 2009, the first case from Mali was reported in a traveler living in southern Mali [10] whiles Ghana reported its first cases amongst two locals living in the Ashanti region in October and December of 2011 [11]. Apart from these two fatal sporadic cases in late 2011, there have been no reports of imported cases from neighboring endemic countries especially Liberia, where Ghana has been contributing Peacekeeping troops since the onset of the crisis in the early 1990’s. Although travellers to endemic countries are at risk of infection [7, 12–15], few outbreaks have been reported among UN Peacekeepers [16]. This paper reports the first two imported cases of Lassa fever among Ghanaian UN Peacekeepers in rural Liberia, who became ill while in country and were subsequently evacuated to the United Nations (UN) level IV hospital in Accra, where their illnesses were laboratory confirmed. With the detailed laboratory investigation and molecular characterization of the virus described [17], this case report discusses the clinical and epidemiologic investigations, the surveillance and management of contacts undertaken following these confirmed cases of imported Lassa fever.

## Case Presentation

### Case 1

A 32 year old male Ghanaian soldier on UN peacekeeping mission in Liberia who resided under remote rural conditions at a new position in Zorzor, Lofa County, in Northwestern Liberia for three and half months, reported to the Company Aid Post (CAP) on 15th May, 2013 with complains of fever, chills, malaise and headaches. He was started on empirical treatment for malaria and then referred to the UN Level 1 hospital for further evaluation based on the suspicion of a possible viral hemorrhagic fever considering the clinical course of a previous case with who he shared the same living accommodation. The index case had died 4 days earlier with hemorrhagic signs following a febrile illness for which he was being managed as a case of confirmed malaria and a possible enteric fever. After 3 days at the level 1 hospital being managed as a case of confirmed malaria, his fever persisted and he also started passing dark urine. He was then referred and admitted at the UN level II hospital on the 22nd May, 2013 for further evaluation and management. He was managed as a case of acute renal failure due to black water fever (severe *Falciparum* malaria). Patient’s condition however failed to improve with a progressively deteriorating renal and liver function as well as a low platelet count. He was thus air-lifted using an air ambulance in a critical condition from Liberia to the 37 Military Hospital in Accra, Ghana on the 26th of May 2013 at around 23:00 h. On arrival the patient was alert and able to give a clear report of his medical history complaining however of vomiting and severe epigastric pain.

Physical examination showed a very ill looking young man who was fully conscious but tachypnoeic (respiratory rate: 36 cycles per minute), febrile with axillary temperature of 38 °C, not pale but with a tinge of jaundice and mildly dehydrated. His blood pressure was 120/90 mmHg and his pulse rate was 68 beats per minute. The chest examination was unremarkable. However, there was moderate general abdominal tenderness with no rebound tenderness. Bowel sounds were present and of normal frequency. An initial diagnosis of viral haemorrhagic fever, probably Lassa fever to rule out septicaemia was made.

Initial laboratory results showed haemoglobin of 12.8 g/dL, white cell count of 44.2 × 10^9^/L and a platelet count of 695 × 10^9^/L. His ESR was 17 mm/h. Blood film for malaria parasite was negative. His renal function was impaired with urea of 27.5 mmol/L and creatinine of 637ummol/L. His electrolytes were however normal. His liver function was also impaired with total bilirubin of 50 μmol/L and AST and ALT of 1321 IU/L and 1060 IU/L respectively. Blood samples were sent to the Noguchi Memorial Institute for Medical Research (NMIMR) for laboratory testing of typical Viral Haemorrhage Fevers (VHFs) the same night.

Application of Reverse Transcriptase-Polymerase Chain Reaction (RT-PCR) assay on blood sample confirmed the presence of Lassa fever virus on 27th May, 2013. The patient was commenced on ribavirin regimen; 30 mg/kg IV (maximum, 2 g) loading dose to be followed by 17 mg/kg IV (maximum, 1 g/dose) 6 hourly for 4 days, then 8 mg/kg IV (maximum, 500 mg/dose) 8 hourly for 6 days in addition to other supportive treatment. Despite intensive nursing and medical care, the patient’s condition deteriorated rapidly on the second day of admission and was later pronounced dead at 17:35 h on the 27th May, 2013. No post-mortem examination was undertaken. Blood culture results received later did not show any bacterial growth.

### Case 2

A 27 year-old male soldier on the same air ambulance as Case 1 from Liberia also arrived at the 37 Military Hospital on the 26th of May 2013 with a 5 days history of fever, headache and 3 days history of diarrhea, vomiting, severe epigastric pain and passage of dark urine. His urine output had also deceased over the period. He shared living accommodation with case 1 and the index case at their position in Zorzor, Lofa County, Liberia and was also on admission at the UN Level II Hospital in Liberia where he was also being managed as a case of Black water fever due to severe *falciparum* malaria.

Physical examination showed an ill-looking young man who was not pale or jaundiced but febrile with a temperature of 39.2 °C. His pulse rate was 72 beats per minute with a blood pressure of 110/70 mmHg. His respiratory system was normal but had marked epigastric tenderness. His central nervous system was grossly intact. An initial diagnosis of complicated malaria with gastritis to rule out Lassa fever was made. Blood sample was also sent to NMIMR for confirmation of suspected viral hemorrhagic fever using RT-PCR. Initial laboratory investigations showed reduced hemoglobin of 9.3 g/dL, low platelet count of 5.5 × 10^9^/L, and white cell count of 5 × 10^9^/L. Blood film for malaria parasites was negative. His urea was 8.5 mmol/L and creatinine was 136 mmol/L, his electrolytes were normal. His liver function test and chest x-ray were normal. Results received on 27th May, 2013 from NMIMR confirmed Lassa fever infection. Blood culture did not grow any organism. Patient was commenced on IV ribavirin 2 g loading dose, then 1 g 6 hourly for 4 days followed by 500 mg 8 hourly for 6 days alongside other supportive treatment.

The patient’s urine output improved within the first week of admission and his epigastric pain gradually resolved. He however continued to have loose stools and occasional vomiting. During the second week of admission he was transfused with four pints of packaged cells as his hemoglobin dropped to 6.8 g/dL with evidence of hemolysis. The patient gradually regained his strength and completed 10 days of ribavirin. Supportive treatment with appropriate nutrition continued until he was discharged without any obvious sequelae of Lassa fever infection after 22 days of admission in the hospital with instructions to avoid unprotected sexual intercourse for 2 months to reduce the risk of transmission of the virus through sex.

### Public health response and management of contacts

In view of previous reported outbreaks of Lassa fever in Liberia [18], there was a high index of suspicion for the imported cases. Consequently, In the UN level IV hospital in Accra, the recommended barrier nursing techniques were fully deployed to receive and admit the patients into the holding rooms. The patients were isolated and managed with strict infection control measures including full body protection using personal protective equipment. There were three levels of decontamination before any medical personnel were allowed to attend to the patient(s) with full protective measures including the use of N95 respiratory masks, disposable protective body gowns, shoe covers, eye goggles, headgear, and gloves. All medical waste from the ward was incinerated daily. Attending medical, nursing, pathology and allied health professionals who were categorized as low risk were made to monitor their own body temperature and report this to the disease control officer by noon each day for 21 days following their last contact with a case or specimen. None of the personnel developed a fever or any other symptoms suggestive of Lassa fever over the observation period.

Facts about the cases were communicated to relevant local, national and international authorities including the World health Organization (WHO), the United Nations Mission in Liberia (UNMIL) Headquarters and the air ambulance crew. The WHO was informed under the International Health Regulations [19], through the WHO Country Office, while the Ministry of Health was notified through the disease surveillance unit of the Ghana Health Service (GHS). The GHS responded appropriately to media inquiries, confirming that there was no risk to the general public resulting from the case. Health talks on Lassa fever were organized for the troops in Liberia and information sheets on Lassa fever and the process of monitoring were made available to the contacts and remaining contingent in Liberia.

All individuals with potential direct exposure to Lassa virus through contact with any of the three cases or exposure to bodily fluids required risk assessment. A five-member Ghanaian medical team from the Battalion headquarters in Liberia headed by a senior medical officer was assisted by the UNMIL medical unit and the public health division of the Level IV hospital in Ghana, to begin control measures in the Zorzor camp through identification and categorization of personal contacts of the cases, institution of daily temperature surveillance for isolated close personal contacts and improving general sanitation and hygiene in the camp. All contacts were assigned to one of three categories depending on their level of risk as no risk, low risk and high risk as reported elsewhere [10] and managed accordingly. The 11 soldiers who shared living accommodation with the 3 cases were classified as potential high risk and evacuated to a new room and quarantined for 21 days with twice daily temperature surveillance and monitoring for signs consistent with Lassa fever. The medical team donned the appropriate personal protective equipment (PPE) and observed standard infection prevention and control measures. The remaining 66 soldiers who either had no direct contact or only casual contact with the cases were informed of the minimal risk and were provided with adequate information about Lassa fever and advised to monitor their own temperature and report any fever to the medical team for further evaluation. Movements into and out of the camp were also restricted. Samples were later taken from the 11 isolated close contacts and the 5 member onsite medical team by the UNMIL medical unit’s investigative team for testing. Results from the enzyme-linked immunosorbent assays (ELISA) performed on the samples detected anti-Lassa fever IgM and IgG antibodies in 4 out of the eleven close contacts. These four new cases were further isolated and commenced on a course of IV ribavirin for 10 days. None of the isolated close-contacts or those who subsequently tested positive showed any overt signs of Lassa fever and were thus discharged after completing the 21-day follow up period. All international contacts including the UN air ambulance crew and the Level II medical team were followed up by the UNMIL medical unit’s incident control team and appropriately managed.

## Discussion

This report describes the first cases of imported Lassa fever diagnosed in Ghana as well as the first hospitalized Lassa fever case in Ghana to survive the disease with close follow up of contacts. The first two indigenous cases diagnosed in the Amansie West district in Ghana both resulted in fatalities [11]. The non-specific presentation of Lassa fever often makes clinical diagnosis in the absence of a high index of suspicion difficult. In the cases described here, the reported confirmed diagnosis of malaria obviously delayed the consideration of other endemic diseases like Lassa fever as a potential cause for the febrile illness. It is however, well established that malaria and Lassa fever may coexist in the same individual [20]. A confirmation of malaria and a developing renal impairment in the two imported cases may have informed the diagnosis of black water fever. It is noteworthy however that, while black water fever in adults from hyper-endemic malaria regions is uncommon, acute renal impairment in Lassa fever patients is not [21]. With the backdrop of previous reports of Lassa fever in Zorzor [18, 22], the characteristic manner in which the index case’s illness progressed to bleeding and the epidemiological link of the two subsequent cases to the index case led to a high index of suspicion of Lassa fever among the receiving Ghanaian medical team. Additionally, the time to onset of the febrile illness in the two imported cases with the characteristic incubation period for Lassa fever also alerted the Ghanaian medical team to deploy the appropriate barrier precautions, isolation and prompt diagnosis of Lassa fever in the imported cases. Tissue samples of the first case who died in Liberia were later sent to NMIMR after the two cases who shared accommodation with him were confirmed to have acquired Lassa fever. Immunohistochemistry and PCR assay performed on the tissue samples later confirmed Lassa fever and made him the missed index case.

Although the risk of human-to-human transmission of Lassa fever is low, nosocomial transmission in the healthcare environment has occurred in some endemic areas [18, 22, 23] and an instance of asymptomatic seroconversion has been reported in a German physician [12]. In these cases described, strict adherence to standard universal infection control practices to prevent unprotected exposures to blood or other body fluids resulted in successful case management with no secondary cases among medical personnel or visitors. With limited proven effectiveness and tolerability of oral ribavirin prophylaxis [24], it was not recommended for persons who might have been exposed to the cases described in this report. Instead, a standard 10 days treatment regimen of intravenous ribavirin was recommended for the 4 out of the 11 soldiers who shared living accommodation with the cases and showed asymptomatic seroconversion for Lassa fever. After strict temperature surveillance and monitoring, none of the contacts including the 7 other soldiers who had some form of close contact with very ill cases developed an illness consistent with Lassa fever.

Field investigation at the Zorzor camp confirmed rodent activity as well as a hole in the floor of the room where the infected cases lived. This finding indicated how the soldiers in that room may have been exposed to the Lassa virus which is shed in the urine and droppings of the *Mastomys* rodents and transmitted through direct contact with contaminated objects or food [1]. After fumigation of the room and repair of the broken floor, other measures were instituted to prevent rodents from entering the inhabited areas. These included rodent traps, storage of food and grain in rodent-proof containers, proper waste disposal and the maintenance of general camp environmental hygiene. These measures as well as continued awareness have been successful so far in preventing another outbreak amongst the peacekeepers.

## Conclusion

In a globalized world where nations and economies are increasingly interdependent with vanishing trade restrictions and geographic boundaries, there is easy movement and travel of humans and goods. The consequence is the potential for rapid transfer of diseases from one geographic region to the other and the likely events of epidemics and outbreaks. Importation of emerging infections such as Lassa fever and other VHFs into non endemic regions is a growing threat especially as international travel and commitments in resolving conflicts in endemic countries in the West Africa sub-region persist. Healthcare providers should thus have a high index of suspicion for VHF and adopt standard infection control measures when treating patients in endemic regions or returning travelers from an endemic region with a febrile illness even of a known etiology. Surveillance of travel-associated diseases will be important to provide the evidence for both pre- and post-travel advice and interventions. In the event of an outbreak with a dangerous pathogen such as the Lassa virus, adopting an appropriate public health response [24] and adhering to strict infection control measures in providing care will help curb the spread and prevent an epidemic.

## Consent

Written informed consent was obtained from the surviving patient for publication of this case report. For the deceased case, consent for publication was sought from the next of kin (wife) of the patient. A copy of the written consents are available for review by the Editor of this journal.

## Abbreviations

ALT: Alanine transaminase
AST: Aspartate transaminase
CAP: Company aid post
ELISA: Enzyme-linked immunosorbent assays
ESR: Erythrocyte sedimentation rate
IgG: Immunoglobulin-G
IgM: Immunoglobulin-M
IV: Intravenous
NMIMR: Noguchi Memorial Institute for Medical Research
PCR: Polymerase chain reaction
PPE: Personal protective equipment
RNA: Ribonucleic acid
RR: Respiratory rate
RT-PCR: Reverse Transcriptase- Polymerase Chain Reaction
UN: United Nations
UNMIL: United Nations Mission in Liberia
VHFs: Viral hemorrhagic fevers
WHO: World Health Organization
